# Management of organ motion in scanned ion beam therapy

**DOI:** 10.1186/s13014-017-0911-z

**Published:** 2017-11-06

**Authors:** Christoph Bert, Klaus Herfarth

**Affiliations:** 1 Department of Radiation Oncology, Universitätsklinikum Erlangen, Friedrich-Alexander-Universität Erlangen-Nürnberg, Universitätsstraße 27, 91054 Erlangen, Germany; 2 Heidelberg Ion-Beam Therapy Center (HIT) and Department of Radiation Oncology, University Clinic Heidelberg, Heidelberg, Germany

**Keywords:** Organ motion, Lon beam therapy, Beam scanning

## Abstract

Scanned ion beam therapy has special demands for treatment of intra-fractionally moving tumors such as lesions in lung or liver. Interplay effects between beam and organ motion can in those settings lead to under-dosage of the target volume. Dedicated treatment techniques such as gating or abdominal compression are required. In addition 4D treatment planning should be used to determine strategies for patient specific treatment planning such as an increased beam focus or the use of internal target volumes incorporating range changes.

Several work packages of the Clinical Research Units 214 and 214/2 funded by the German Research Council investigated the management of organ motion in scanned ion beam therapy. A focus was laid on 4D treatment planning using TRiP4D and the development of motion mitigation strategies including their quality assurance. This review focuses on the activity in the second funding period covering adaptive treatment planning strategies, 4D treatment plan optimization, and the application of motion management in pre-clinical research on radiation therapy of cardiac arrhythmias.

## Background

Up to now one great challenge for scanned particle beam therapy is the management of intra-fractional organ motion since interplay effects between scanned beam and the moving organ can lead to deviations in the dose deposited to the clinical target volume (CTV) [1, 2]. The focus of several work packages of the Clinical Research Units (KFO) 214 and 214/2 funded by the German Research Council (DFG) was therefore related to methods and devices aiming at a safe and reliable treatment of moving organs with a scanned beam. In the following the main findings of the second funding period are summarized focusing on 4D treatment planning with an emphasis on 4D treatment plan optimization, its application for determining parameters in adaptive ion beam therapy and for pre-clinical research addressing cardiac arrhythmias. This review is by definition very biased towards the work of the funded groups. More general reviews on that topic can be found in the literature [3–7].

## 4D treatment planning

Within the pilot project of the GSI Helmholtz Centre for Heavy Ion Research in Darmstadt, Germany (GSI) on carbon beam therapy all patients’ treatment was planned with “Treatment planning for particles” (TRiP98) as initially reported by Krämer et al. [8, 9]. Starting in 2002, TRiP98 was extended by the time domain to address 4D treatment planning (TRiP4D) [10–16]. The software has interfaces to the GSI therapy control system (TCS) for 4D optimized treatment plans (see next section) and accepts treatment delivery log files from the beam application systems of GSI and the Heidelberg Ion-Beam Therapy Center (HIT) for calculation of the 4D delivered dose. TRiP4D is not certified for clinical use and thus remains a research tool. Apart from the applications in research studies (see below on animal experiments addressing cardiac arrhythmia) TRiP4D was used to explore treatment parameters for patients with hepato cellular carcinoma (HCC) which are treated at HIT according to the protocol of the PROMETHEUS-01 trial [17].

## Treatment parameters and 4D dose reconstruction for HCC therapy

Richter et al. determined by a series of 4D dose calculations using the data of 8 HCC patients which parameters reduce best the residual interplay in treatments based on gating or abdominal compression [18]. They found that an increased pencil beam size has the biggest effect and that similar motion amplitudes yield comparable V95 target coverage for patients treated under abdominal compression. Currently, HIT uses pencil beams with a FWHM of 10 mm at 2 mm raster spacing to treat those tumors. For the first patients with HCC treated at HIT with a scanned ion beam, Richter et al. reconstructed the daily dose delivery using the beam delivery sequence and log files from the ANZAI system used for motion detection [19]. In addition to delays in the gating sensors [20] the technical implementation resulted into a temporal uncertainty between the log files of beam delivery sequence and motion detection. The dose reconstruction analysis found that 25 ms temporal uncertainty can be permitted for an acceptable accuracy in the reconstructed V95 of the clinical target volume (CTV). HIT therefore meanwhile implemented an improved data acquisition system. Despite these limitations the feasibility of daily 4D dose reconstruction could be shown but is not yet part of daily clinical workflows. A general limitation is the choice of the CT data underlying all calculations incl. deformation maps connecting the phases of the 4DCT. Typically these data are not acquired at time of treatment such that inter-fractional changes influence the accuracy of the calculations. Dose calculations based on CBCT recently reported by other groups are possible solution to overcome that issue [21, 22].

## Modelling of 4DCT data

One option to overcome those limitations is modelling of the daily 4DCT. The extension of such a model and its test against clinical data was reported by Wölfelschneider et al. for lung cancer [23]. The model uses the treatment planning 4DCT to calculate vector fields from the mid-ventilation image to each motion phase of the 4DCTs. In order to generate a daily 4DCT, imaging data from patient positioning such as a CBCT are used to determine the baseline shift of the tumor and motion surrogates such as the contour of the patient’s skin (which could e.g. determined by surface imaging) are used to determine the varying phase and amplitude [24] of the intra-fractional motion. The modelled 4DCTs were checked against regular follow-up 4DCTs from clinical data. Differences of (61.3 ± 16.7) HU were determined. In addition, approximately 400 scale invariant features were extracted from the modelled as well as from the ground-truth 4DCT. Median distances of (2.9 ± 3.0) mm were determined between those landmarks. The authors concluded that the modelled 4DCTs can be used for 4D dose calculations in photon therapy. For the same model Fassi et al. determined changes in water equivalent path-length of <1.9 mm [25].

## 4D optimized treatment planning

4D optimized treatment planning uses time as an additional degree of freedom [26, 27]. It can serve as a motion mitigation technique itself or be used in combination with other techniques such as gating, rescanning, or beam tracking to improve the treatment delivery. In those cases often a dedicated treatment control system is necessary such that organ motion, i.e. the breathing phases determined by a motion monitoring system, is incorporated into the delivery process. A review on the methodologies was reported by C. Graeff [5].

To a certain extent, time was already included in the early reports on tracking with a scanned ion beam since a look-up table (LUT) was required to provide the motion compensation parameters for all combinations of raster points/beam positions and motion phases [12, 28]. In that approach target motion was handled in the treatment plan generation process but not directly in the optimization. That requires summation over the different motion phases in the cost function which in general can be described as

$$ E\left(\overrightarrow{N}\right)=\sum \limits_{k=1}^m\sum \limits_{i=1}^v{\left[{D}_{pre}^i-{D}_{act}^{ik}\left({\overrightarrow{N}}_k\right)\right]}^2=\sum \limits_{k=1}^m\sum \limits_{i=1}^v{\left[{D}_{pre}^i-\sum \limits_{j=1}^r{c}_{ijk}{N}_{jk}\right]}^2 $$

with *D*_*pre*_ the prescribed dose and *D*_*act*_ the actual dose during the optimization process for voxel *i*, *N* the particle number delivered to beam spot *j* during motion phase *k*. The difference to 3D optimization is a number of motion phases *m* > 1 allowing that, e.g. an organ at risk (OAR) with motion related changes in distance to the planning target volume (PTV) will get favorable scores in the distant motion phases once OAR burden is incorporated in the cost function by additional terms.

Eley et al. implemented that approach for beam tracking [29]. The approach was assessed in simulations for simple geometries and lung cancer cases considering absorbed dose, only. In case of phantoms the dose to an OAR could be reduced by 53%, for patient data sets by 13%, each relative to 3D beam tracking. Target coverage was similar for the phantom and significantly improved for the patient. A full feasibility check also requires proof for deliverability. Eley et al. thus also upgraded the GSI TCS such that tracking was delivered in combination with gating, i.e. individual motion phases of the 4D treatment plan were delivered sequentially. The experiment used phantoms with a simple target/OAR setup showed the expected reduction in dose to the OAR (mean optical density of the film in the OAR reduced from 0.71 to 0.26 going from 3D to 4D tracking) but required very long irradiation times.

A similar strategy was addressed by Graeff et al. [16] but extended to RBE-effective dose and applying a strategy that resulted in faster delivery. The main idea was sector-wise division of the target volume into multiple 3D treatment plans all on a single grid of beam positions. Each of the plans corresponded to one motion state and all of them were optimized in 4D in parallel. Also Graeff et al. required a dedicated 4D TCS with gating functionality but the delivery process was more efficient than described before: during the application the beam scans along the single grid underlying all 3D treatment plans corresponding to the individual motion phases. A motion monitoring signal determines the motion phase and the particle numbers of the corresponding 3D treatment plan are delivered. As long as all individual 3D treatment plans contain beam positions in a delivered iso-energy slice (IES), delivery is continuous. Once certain 3D plans applied all positions of an IES the beam is gated in the corresponding motion phase. The approach was successfully implemented as shown in irradiations of radiographic films. Simulations using data of 9 lung cancer patients resulted in target coverages slightly lower than the stationary optimizations (median V95 of 97.9% and 99.3% for 4D–optimized and stationary, respectively).

The reported studies on 4D optimized treatment planning are at a very early stage, i.e. based on simulations and experimental studies involving phantoms. For clinical application further developments especially with respect to (commercialized) 4D treatment planning systems and 4D treatment control systems are essential.

## Adaptive ion beam therapy

Adaptive treatment planning aims at improved target conformation by changing the treatment plan on a daily level such that the dose delivery matches best to the varying anatomy of the patient. Clinical examples include treatment of bladder cancer [30], prostate cancer [31], or lung lesions [32]. In several reports, a reduction in normal tissue dose at comparable target coverage is reported.

One reason that adaptive treatment schemes are currently not widely adopted in ion beam therapy could be the impracticability of frequent changes of patient specific hardware such as compensator or collimator in therapy centers using passive scattering. More recently established centers typically rely on beam scanning which does not bring such hardware limitations but currently neither use adaption of treatment plans on a widespread level. In the few reports on treatment plan adaption online adaptation is typically done either using a library of treatment plans optimized e.g. based on multiple (CB)CT data of the first treatment fractions (plan-of-the-day approach) [33, 34] or by swift re-optimization of the treatment plan based on daily imaging data [35, 36].

For prostate cancer treatments using carbon beam therapy Hild et al. studied in a treatment plan comparison three different treatment approaches, namely conventional therapy using a single plan and geometrically defined margins, an offline approach using a varying number of CT datasets to form an internal target volume (ITV), and an online approach including daily re-optimization of the treatment plan [37]. They determined that adequate CTV coverage can be assured with all studied concepts if the prostate motion is below 4 mm. For larger motion, only the online approach resulted in a V95 of the CTV >95%. The offline approach with 4 datasets and especially the online approach with its reduced margins resulted in parallel in a significant reduction of the dose deposited in bladder and rectum. As also authors from other studies [38, 39] reporting online ART with treatment plan re-optimization Hild et al. stress the increased daily workload (in particular re-contouring), the computational load for the daily optimization + dose calculation, and the development of adequate quality assurance. Due to parallelization of TRiP4D the time demands could be decreased to ~ 6 min which might already be sufficiently short for a clinical application [40].

With respect to the need for treatment plan adaptation due to dosimetric influence of inter-fractional changes in lung cancer treatments, Brevet et al. used serial 4DCTs to investigate in a treatment plan comparison the target coverage in gated scanned ion beam treatments [41]. Based on 9 data sets containing 6–10 weekly 4DCTs per patient a single treatment plan was optimized based on the first 4DCT. Plan optimization used the ITV approach of Graeff et al. [15] to cover residual motion in the gating window of 25% of the breathing motion amplitude. For varying parameters of gating window and size of the beam focus, the coverage of the target (V95) and the conformity index were investigated by repeated dose calculations based on the weekly 4DCTs. In addition, the number of fields of the treatment plan was varied since a homogenization effect is expected [42], and the ITV was expanded by additional margins. Calculation of the 4D dose distributions using TRiP4D considered the inter- and intra-fractional motion component. The results show that a combination of increased beam focus size (15 mm FWHM), reduced gating window (11.9%), additional ITV-PTV margins especially addressing the beam range, and dose application by 3 fields yielded the best target coverage of the multi-week fractionated treatment scheme. A V95 coverage of the CTV of 96.5% was determined for that treatment parameter combination.

## 4D treatment planning for the treatment of cardiac arrhythmias

Cardiac arrhythmias and especially atrial fibrillation (AF) as the most common arrhythmia [43] is a major cause of stroke [44] and effects more than 2.3 million patients in the USA per year [45]. In patients suffering from AF the sinus rhythm of the heart is disturbed due to disorganized electrical impulses originating from the pulmonary veins or the atria which occur in parallel to the impulses of the sinoatrial node, the natural pacemaker. This leads to an irregular accumulated impulse to the ventricles that causes the dangerous irregular heart beat and predisposes stroke. To date, standard of care to treat these patients is electrical isolation of the pulmonary veins by endocardial radio frequency ablation (ERFA) or drug therapy [44]. ERFA is an invasive and typically more than 5 h long intervention [46] with only 75% success rate after 1 year [47]. Besides required improvements of success rates, reduction of severe complications is essential since these affect 6% of the patients and include peri-procedural death and stroke [47]. Last but not least treatments of AF are very expensive, leading to annual cost of €13.5 billion in the EU [48].

Initial studies indicate that radiation therapy might be a non-invasive alternative to ERFA [49, 50]. These studies were conducted with photon beam therapy and thus suffer from a lower target conformation and especially higher integral normal tissue dose than particle beam therapy. In a joint project of GSI, the Heidelberg University Clinic in Heidelberg, Germany and the Mayo Clinic in Rochester, Mn USA the feasibility of carbon beam therapy of cardiac fibrillation has been tested in an animal study after in-vitro irradiations of an explanted heart in a Langendorff setup showed that AV blocks can be achieved if sufficiently high doses are applied [51, 52].

All details of the conducted study are reported by Lehmann et al. [53]. In total 17 pigs were randomized for either irradiation of the AV node (*n* = 8), the right superior pulmonary vein left atrial junction, the left ventricle and to comparable sham-procedures (each *n* = 3). Irradiation was delivered at GSI using rasterscanned pencil beams on a horizontal beam line. A dose of 40 Gy in a single fraction was delivered to the target volume apart for the AV node group, were three different dose levels (25 Gy (*n* = 2), 40 Gy, 55 Gy (each *n* = 3)) were delivered.

Scanned particle beam delivery to the beating heart is influenced by cardiac as well as respiratory motion. To compensate the dosimetric influence of respiratory motion the ventilated and sedated animals were treated in end-exhale for all therapeutically relevant procedures (imaging, positioning, irradiation) by controlling the respirator such that a 25 s breath-hold at end-exhale was achieved. Influence of cardiac motion was addressed by rescanning and assessed by 4D treatment planning using TRiP4D as described above. 4DTP started with deformable image registration for propagation of contours and 4D dose calculation. The core part were multiple 4D dose calculations to investigate the influence of changing breathing and delivery parameters on the dose distribution. Similar studies were performed by Constantinescu et al. to study the feasibility of AF treatments for humans [54]. Within that approach margins (lateral and range) and the number of rescans were optimized such that the clinical goals were met. 15 rescans in the distal slices resulted in a sufficiently homogenous target coverage. Prior animal irradiation the treatment plans were delivered into a water tank using the robotic 4D phantom developed by Steidl et al. [55]. During delivery of these treatment plans and especially during treatment of the animals, the log files of the beam delivery sequence and the ECG trace were recorded such that the delivered 4D dose could be reconstructed [56].

Treatment outcome was judged after 24–40 weeks using among other tests electroanatomical mapping against baseline data and inspection of the macroscopic lesion. The data show that scanned carbon beam therapy allows chronic interruption of impulse propagation in the heart 13–17 weeks after a single irradiation with 40–55 Gy. No severe radiation induced side effects were seen. Concerning 4D reconstruction of the delivered dose using TRiP4D, Richter et al. showed that within 30 min after irradiation a preliminary dose assessment of individual fields was possible [56]. Target volume D95 dose levels were >95% in all but one animal for which technical reasons could be identified to explain the deviation. OAR dose differed in median by 0.1% from the planned dose. The workflow was initially developed for HCC treatments at HIT [19] but improvements allowed dose assessment briefly after dose delivery, a prerequisite, e.g. for adaptive treatment schedules.

## Conclusions & outlook

Within the scope of the funded period an infrastructure was set up at GSI/HIT allowing to investigate several relevant issues concerning the management of organ motion in scanned ion beam therapy. A central item is TRiP4D, an in-house 4D treatment planning system, which allows treatment plan optimization and dose calculation in the presence of motion. Within that period simulation and experimental phantom studies on 4D optimized treatment plans and simulation studies addressing the dosimetric benefits of adaptive treatments have been performed. In addition, first patients with intra-fractionally moving hepato cellular carcinoma have been treated with a scanned carbon beam at HIT using parameters identified in pre-clinical research based on 4D dose calculations and irradiations using in-house developed motion phantoms. Treatment outcome was successfully monitored by reconstructing the delivered dose distribution. Recently, preclinical research on charged particle beam treatment of cardiac arrhythmias was based on 4D treatment planning and delivery using the GSI infrastructure.

Future investigations will lead to ion treatment of pancreatic cancer und lung cancer using a scanned beam. Tumor movement will probably have a stronger impact on the dose distribution in a target surrounded by low density tissue compared to targets in the liver.

## Abbreviations

4D: Four dimensional
AF: Atrial fibrillation
AV node: Atrioventricular node
CBCT: Cone beam CT
CT: Computed tomography
CTV: Clinical target volume
D95: Minimal dose, covering 95% of the volume of interest in the dose volume histogram
DFG: German research council
ERFA: Endocardial radio frequency ablation
EU: European union
FWHM: Full width at half maximum
GSI: GSI Helmholtz centre for heavy Ion research in Darmstadt, Germany
HCC: Hepato cellular carcinoma
HIT: Heidelberg Ion-beam therapy center
HU: Hounsfield unit
IES: Iso-energy slice
ITV: Internal target volume
KFO: Clinical research unit
LUT: Look-up table
OAR: Organ at risk
PTV: Planning target volume
TCS: Therapy control system
TRiP4D: extension of TRiP98 by the time domain
TRiP98: Treatment planning for particles
V95: Fraction of the dose volume histogram covered with more than 95% of the prescribed dose
